# Supplementary material for: High Dose IFN-β Activates GAF to Enhance Expression of ISGF3 Target Genes in MLE12 Epithelial Cells
Source: Front Immunol. 2021 Apr 9;12:651254. doi: 10.3389/fimmu.2021.651254 (PMC8062733; doi:10.3389/fimmu.2021.651254)
Supplement: Supplementary file 1 [file DataSheet_1.pdf]

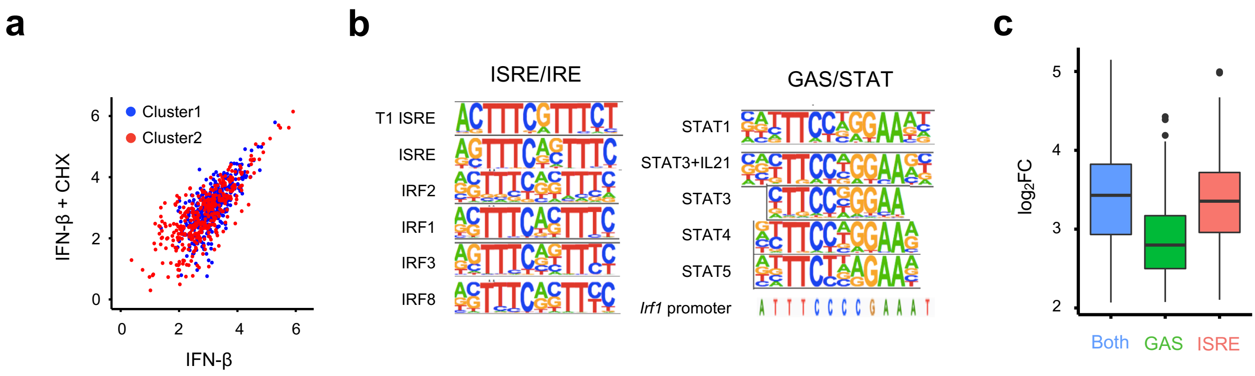

Figure S1: Supplemental STAT1 ChIP-seq data. (a) Scatterplot of STAT1 ChIP-seq signal, $\log_2$ of normalized tag counts for 723 peaks, comparing IFN-β stimulation (10 U/ml) with and without cycloheximide. Blue dots are Cluster 1 peaks, red dots are Cluster 2 peaks as defined by K-means clustering in Fig 1b. **(b)** JASPAR matrices from the HOMER database used in motif-based classification of STAT1 ChIP-seq peaks. **(c)** Box plots of maximum $\log_2$ fold-change over four-hour time course for STAT1 peaks containing BOTH, GAS, or ISRE motifs.

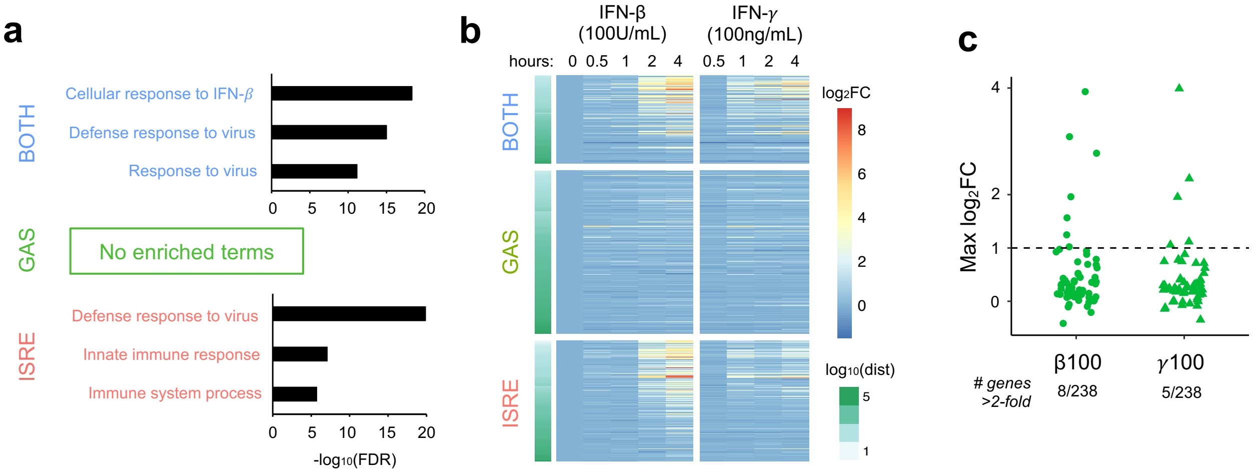

Figure S2: Supplemental characterization of genes linked to GAS peaks. (a) Ontology analysis of genes linked to STAT1 peaks containing Both, GAS, or ISRE motifs. **(b)** Heat map of inducible expression (log_2 fold-change) for genes linked to STAT1 peaks containing Both, GAS, or ISRE motifs in response to very high doses of IFN-β (100 U/ml) or IFN-γ (100 ng/ml) in MLE-12 cells. **(c)** Dot plot of log_2 fold-change for genes linked to GAS peaks, in response to very high dose IFN-β and IFN-γ as in (b). Dotted line indicates 2-fold induction threshold.

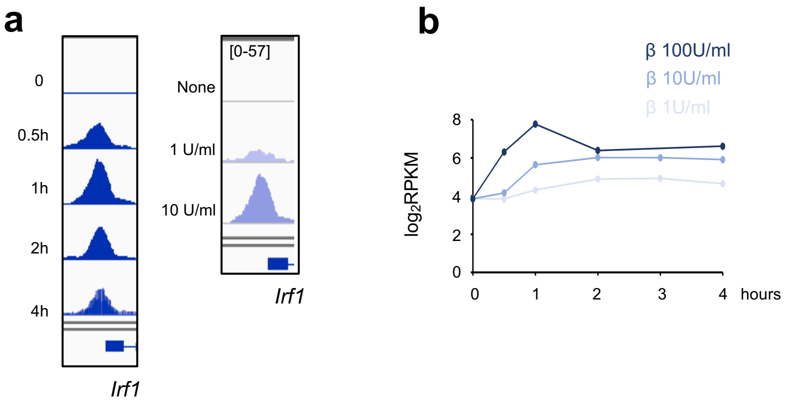

Figure S3: Induction of IRF1. (a) Genome browser tracks of STAT1 ChIP-seq at *Irf1* promoter. Left, time course with 10 U/ml IFN-β. Right, low vs high-dose IFN-β. **(b)** Gene expression of *Irf1* in response to different doses of IFN-β. **(c)** Western blot of IRF1 in MLE12 cells stimulated with IFN-β (10 U/ml) at the indicated time points.

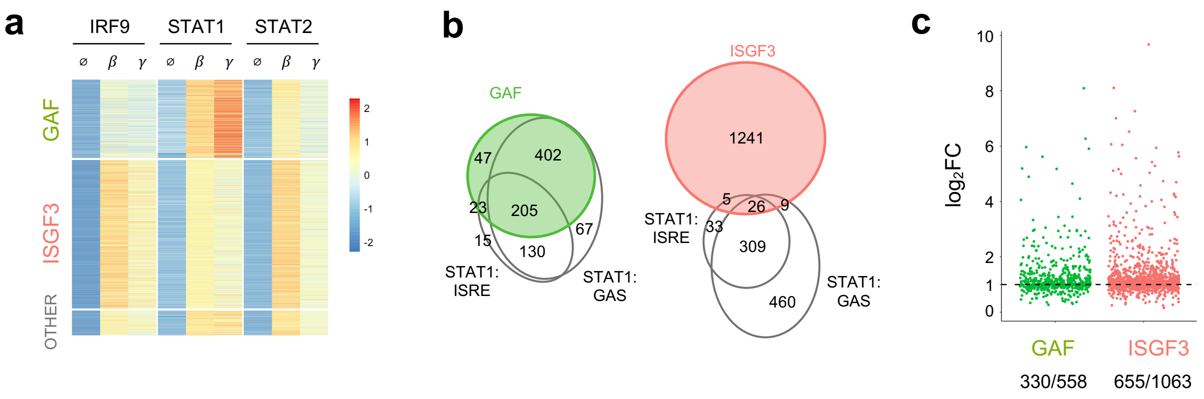

Figure S4: Categorization of BMDM ChIP-seq peaks by TF binding pattern. (a) Heat map of ChIP-seq data in macrophages *(21)*, categorized into GAF and ISGF3 peaks by TF binding pattern. **(b)** Venn diagrams comparing peak categorization by TF binding pattern (as in Fig. S4a, colored circles) *vs.* by motifs in STAT1 peaks (as in Fig. 4, empty circles). **(c)** Dot plot of $\log_2$FC expression of genes linked to peaks categorized by TF binding pattern.

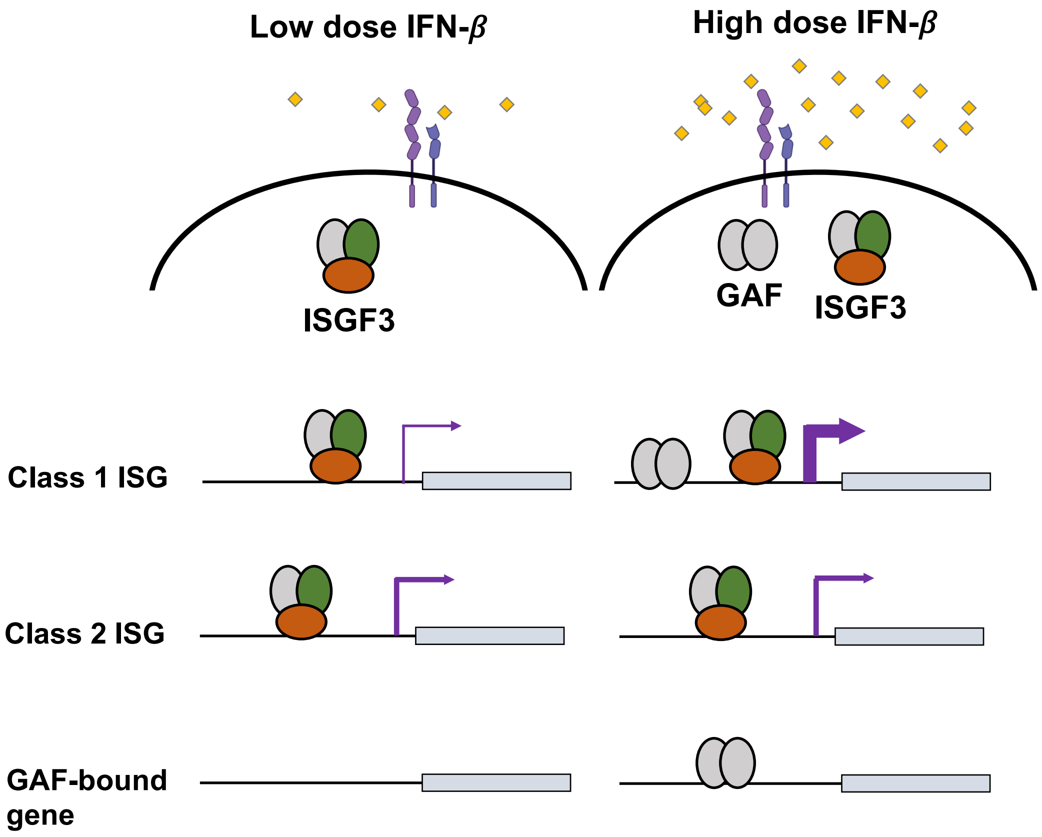

Figure S5: Schematic of proposed model. High but not low doses of IFN-β activate both GAF and ISGF3. ISGs fall into two classes. Expression of class 1 ISGs is enhanced when GAF is active. Expression of class 2 ISGs is not influenced by GAF and is similar in low and high IFN-β conditions. Genes that are bound by GAF alone are not expressed.
